# Role of the protease-activated receptor-2 (PAR2) in the exacerbation of house dust mite-induced murine allergic lung disease by multi-walled carbon nanotubes

**DOI:** 10.1186/s12989-023-00538-6

**Published:** 2023-08-14

**Authors:** Ho Young Lee, Dorothy J. You, Alexia Taylor-Just, Logan J. Tisch, Ryan D. Bartone, Hannah M. Atkins, Lauren M. Ralph, Silvio Antoniak, James C. Bonner

**Affiliations:** 1 Toxicology Program, Department of Biological Sciences, North Carolina State University, Raleigh, NC USA; 2 Department of Population Health and Pathobiology, North Carolina State University, Raleigh, NC USA; 3 Department of Pathology and Laboratory Medicine, University of North Carolina, Chapel Hill, NC USA; 4 Lineberger Comprehensive Cancer Center, University of North Carolina, Chapel Hill, NC USA; 5 UNC Blood Research Center, Department of Pathology and Laboratory Medicine, School of Medicine, University of North Carolina at Chapel Hill, Chapel Hill, NC USA

**Keywords:** Carbon nanotubes, Allergens, Protease-activated receptor 2, Lung, Inflammation, Fibrosis

## Abstract

**Background:**

Pulmonary exposure to multi-walled carbon nanotubes (MWCNTs) has been reported to exert strong pro-inflammatory and pro-fibrotic adjuvant effects in mouse models of allergic lung disease. However, the molecular mechanisms through which MWCNTs exacerbate allergen-induced lung disease remain to be elucidated. We hypothesized that protease-activated receptor 2 (PAR2), a G-protein coupled receptor previously implicated in the pathogenesis of various diseases including pulmonary fibrosis and asthma, may play an important role in the exacerbation of house dust mite (HDM) allergen-induced lung disease by MWCNTs.

**Methods:**

Wildtype (WT) male C57BL6 mice and *Par2* KO mice were exposed to vehicle, MWCNTs, HDM extract, or both via oropharyngeal aspiration 6 times over a period of 3 weeks and were sacrificed 3-days after the final exposure (day 22). Bronchoalveolar lavage fluid (BALF) was harvested to measure changes in inflammatory cells, total protein, and lactate dehydrogenase (LDH). Lung protein and RNA were assayed for pro-inflammatory or profibrotic mediators, and formalin-fixed lung sections were evaluated for histopathology.

**Results:**

In both WT and *Par2* KO mice, co-exposure to MWCNTs synergistically increased lung inflammation assessed by histopathology, and increased BALF cellularity, primarily eosinophils, as well as BALF total protein and LDH in the presence of relatively low doses of HDM extract that alone produced little, if any, lung inflammation. In addition, both WT and par2 KO mice displayed a similar increase in lung *Cc1-11* mRNA, which encodes the eosinophil chemokine CCL-11, after co-exposure to MWCNTs and HDM extract. However, *Par2* KO mice displayed significantly less airway fibrosis as determined by quantitative morphometry compared to WT mice after co-exposure to MWCNTs and HDM extract. Accordingly, at both protein and mRNA levels, the pro-fibrotic mediator arginase 1 (ARG-1), was downregulated in *Par2* KO mice exposed to MWCNTs and HDM. In contrast, phosphorylation of the pro-inflammatory transcription factor NF-κB and the pro-inflammatory cytokine CXCL-1 was increased in *Par2* KO mice exposed to MWCNTs and HDM.

**Conclusions:**

Our study indicates that PAR2 mediates airway fibrosis but not eosinophilic lung inflammation induced by co-exposure to MWCNTs and HDM allergens.

**Supplementary Information:**

The online version contains supplementary material available at 10.1186/s12989-023-00538-6.

## Introduction

Allergic asthma is a major respiratory disease caused by genetic predisposition and environmental exposure to allergens, such as those derived from the house dust mite (HDM) *Dermatophagoides pteronyssinus*. Asthma can be further exacerbated by inhaling various airborne particles, including ultrafine air pollution particulate matter (PM) from natural sources such as wildfires or anthropogenic sources such as coal-fired power plants or diesel exhaust emissions [1–5]. Inhaled particles increase the severity of pathological features of allergic lung disease, such as eosinophilic inflammation, mucous cell metaplasia, and airway fibrosis, as well as physiological manifestations such as airway hyper-responsiveness (AHR). Thus, while inhaled allergens are the primary causes of the asthmatic phenotype, inhaled particles exacerbate the pulmonary pathological and physiological outcomes.

Engineered nanomaterials (ENMs) represent an emerging source of particles that have been shown to exacerbate allergic airway inflammation in experimental animal models and, therefore, could pose a risk for the exacerbation of allergic asthma in humans. Multi-walled carbon nanotubes (MWCNTs) are a prototypical ENM composed of multiple layers of concentric graphene sheets with diameters ranging from 10 to 100 nm [6]. MWCNTs are used in various applications, including catalysts, coatings, electronics, batteries, and additives to polymers and composites [7]. The unique physicochemical properties possessed by MWCNTs are likely to continue to promote and further diversify how they are used, leading to the expansion of applications that could result in increased occupational and environmental exposures. Numerous epidemiological studies have shown a positive correlation between exposure to carbon-based particulate matter and exacerbation of various respiratory pathologies such as respiratory infections, asthma, and chronic obstructive pulmonary disease (COPD) [8–11]. In contrast to ambient air pollution particles, which are complex mixtures that vary widely depending on the source, ENMs such as MWCNTs offer a well-characterized and relatively homogeneous source for investigating particle exacerbation of allergic lung disease. In experimental animal models, pulmonary exposure to MWCNTs produces a strong adjuvant-like effect in combination with common allergens that exacerbate allergic lung diseases [12–15]. For example, the intranasal aspiration of MWCNTs in mice exacerbates allergic lung disease induced by HDM extract [14]. However, the mechanisms through which MWCNTs, or any particle, produce adjuvant-like effects on allergen-induced lung disease have yet to be fully understood.

Protease-activated receptors (PARs), belonging to a family of transmembrane G protein-coupled receptors (GPCRs), are known to be widely expressed on various types of cells in the lung, including alveolar and bronchial epithelial cells, endothelial cells, and leukocytes [16]. PARs are known to be proteolytically cleaved and activated by serine and cysteine proteases, including proteolytically active proteins found in HDM extract [17]. PARs can be viewed as an integral component of the host antimicrobial alarm system since invading microorganisms release various cysteine and serine proteases capable of activating PARs [18]. In addition, host immune cells can also produce proteases to activate PARs, which leads to GPCR-mediated intracellular signaling that can initiate immune responses such as the recruitment of inflammatory cells and activation of fibroblasts [18–20]. A number of challenges in elucidating the precise roles of PARs in immune and inflammatory diseases include variability in cell type-dependent expression levels of PARs, diversity of G-proteins involved, and potential receptor cross-talk capabilities [21]. Nevertheless, PARs represent potential therapeutic targets for treating immune-related diseases, including allergic asthma.

Studies with PAR2 knock-out (*Par2* KO) mice have been valuable in determining the role of this receptor in several lung diseases. For example, PAR2 deficiency reduced bleomycin-induced pulmonary fibrosis in mice, while PAR2-activating proteases induced fibroblast migration and extracellular matrix production [22]. Other work showed that PAR2-deficient mice displayed reduced allergic lung inflammation upon exposure to HDM extract [23]. Furthermore, pharmacologic administration of anti-PAR2 antibodies decreased airway hyperresponsiveness and inflammation in mice challenged with ovalbumin [24]. To our knowledge, the role of PAR2 in the exacerbation of allergic lung disease by inhaled particles has not been investigated. In the current study, we utilized *Par2* KO mice to explore the role of PAR2 in the exacerbation of HDM extract-induced allergic lung disease by MWCNTs. We hypothesized that PAR2 would mediate the adjuvant-like effects of MWCNTs on HDM allergen-induced airway inflammation and fibrosis. We discovered that *Par2* KO mice did not display any change in the exacerbation of HDM-induced eosinophilic lung inflammation by MWCNTs, nor did *Par2* KO mice display any difference in the number or size of MWCNT-induced lung granulomas compared to wild-type (WT) mice. However, *Par2* KO mice exhibited reduced airway fibrosis and a significantly lower expression of arginase-1 (ARG-1) in lung tissue compared to WT mice after co-exposure to MWCNTs and HDM extract, suggesting that PAR2 plays a role in pro-fibrotic events surrounding the airways of the lung.

## Materials and methods

### House dust mite (HDM) extract

HDM extract from *Dermatophagoides pteronyssinus* was purchased from Greer Laboratories Inc. (Lenoir, NC). Lyophilized HDM was dissolved in 0.1% Pluronic in Dulbecco’s phosphate buffered saline (DPBS) to achieve a stock total HDM extract protein concentration of 1 mg/ml. The HDM extract contained 1610 endotoxin units (EU), determined by amoebocyte lysate test, according to the manufacturer. Stock solution was further diluted with 0.1% Pluronic in DPBS to achieve the necessary working concentrations for dosing.

### Multi-walled carbon nanotubes (MWCNTs)

**Fig. 1:**
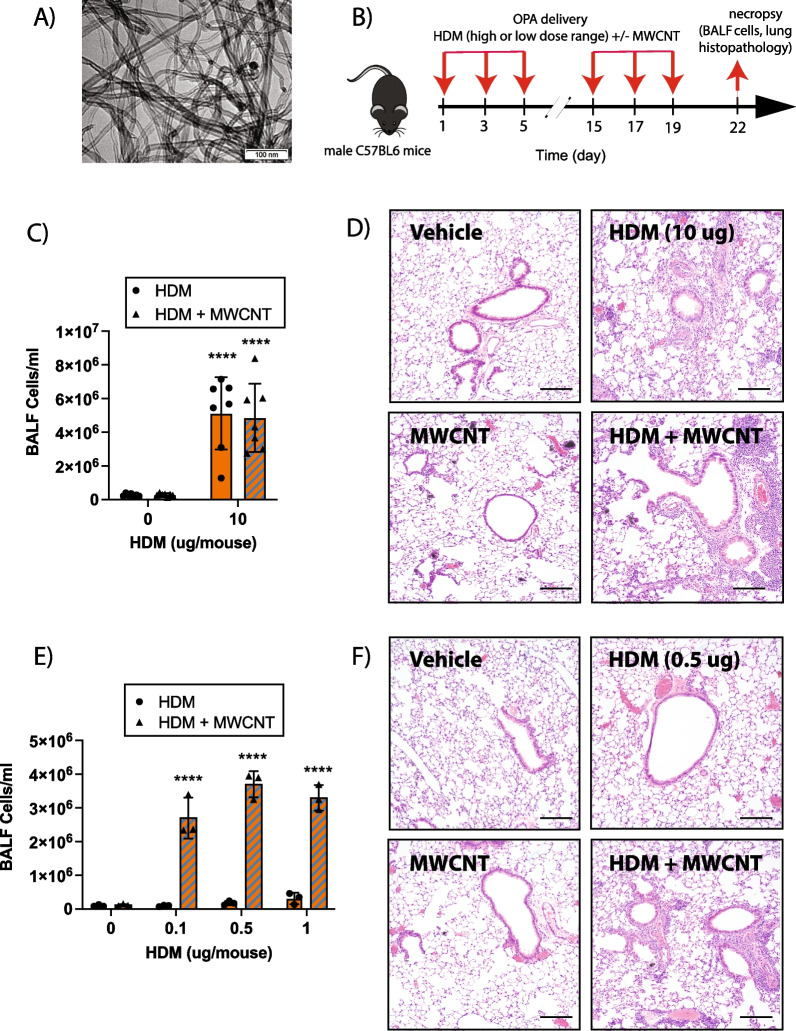
Comparison of high- and low-dose HDM extract on MWCNT-induced lung inflammation in male wildtype (WT) C57BL/6J mice. **A** TEM image of NC7000 MWCNTs used in this study. **B** Graphical representation of the experimental protocol used for Experiment 1 using a high dose of HDM extract (panels **C** and **D**) and experiment 2 using a low dose range of HDM extract (panels **E** and **F**). **C** Total inflammatory cell numbers in the BALF collected from mice exposed to high dose of HDM extract (10 µg per dose equivalent to 0.4 mg/kg per dose) with or without MWCNT (12.5 µg per dose equivalent to 0.5 mg/kg per dose) via oropharyngeal aspiration. n = 7. ****p < 0.0001 when comparing groups with and without MWCNTs as determined by two-way ANOVA with Tukey’s post hoc analysis. **D** Representative images of hematoxylin and eosin-stained lung tissue sections of mice treated with vehicle, high dose of HDM extract, MWCNTs, or combination of both. Black bars indicate 100 µm. **E** Total cell counts in BALF collected from mice exposed to low doses (0.1 µg, 0.5 µg, or 1 µg per dose) of HDM extract with or without MWCNTs (12.5 µg per dose) via oropharyngeal aspiration. n = 3. ****p < 0.0001 when comparing groups with and without MWCNTs as determined by two-way ANOVA with Tukey’s post hoc analysis. **F** Representative images of hematoxylin and eosin-stained lung tissue sections of mice treated with vehicle, low dose of HDM extract (0.5 µg per dose), MWCNTs, or combination of both. Black bars indicate 100 µm

**Fig. 2:**
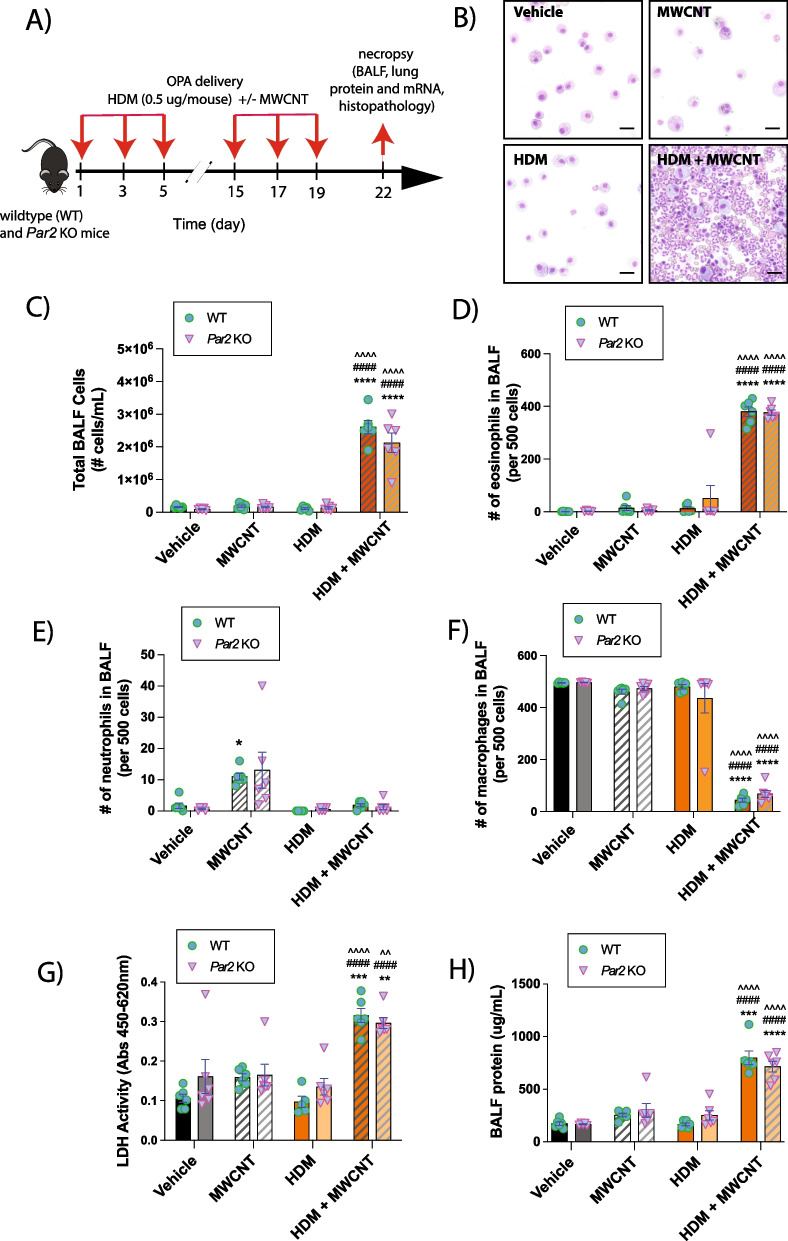
Cellularity analysis and biomarkers of lung injury in BALF collected from WT and *Par2* KO mice exposed to HDM extract and MWCNTs. **A** Graphical representation of the experimental protocol. **B** Representative images of cytospins of BALF from WT mice showing increased eosinophilic inflammation after co-exposure to MWCNTs and HDM extract. Black bars = 10 µm. **C** Total inflammatory cell numbers in the BALF collected from mice exposed to low dose (0.5 µg per dose) of HDM extract with or without MWCNTs via oropharyngeal aspiration. Differential cell counts in BALF expressed as relative number per 500 cells: **D** Eosinophils. **E** Neutrophils. **F** Macrophages. **G** BALF LDH activity. **H** BALF total protein. n = 6. ****p < 0.0001, ***p < 0.0001, **p < 0.01, *p < 0.05 when compared to respective vehicle control groups, ^^^^*p* < 0.0001, ^^*p* < 0.01 when compared to respective HDM groups, and ####*p* < 0.0001 when compared to respective MWCNT groups as determined by two-way ANOVA with Tukey’s post hoc analysis

MWCNTs (NC7000) were purchased from Nanocyl, Inc. (Sambreville, Belgium) and have been thoroughly characterized previously [25]. The physicochemical characteristics of NC7000 are listed in Additional file 1: Table S1. A representative transmission electron microscope (TEM) image of NC7000 is shown in Fig. 1A. MWCNTs were suspended in 0.1% Pluronic F-68 (ThermoFisher Scientific, Waltham, MA) in DPBS (Sigma, St. Louis, MO) to achieve a stock concentration of 10 mg/mL. Pluronic is a non-ionic surfactant used to disperse MWCNTs in aqueous media [25]. Prepared stock of MWCNTs suspension was sonicated in a cup horn sonicator (Q500, Qsonica, Newtown, CT) for 10 min at 60 amps. Stock solution was further diluted with 0.1% Pluronic in DPBS to achieve a working concentration of 0.25 mg/mL for dosing. The dosing strategy for low or high dose HDM extract in the absence or presence of MWCNTs is illustrated in Figs. 1B and 2A.

### Animal care

Wildtype (WT) male C57BL/6 J mice (10 weeks old) and *Par2* KO (B6.Cg-F2rl1^tm1Mslb^/J) male mice (10 weeks old) were purchased from The Jackson Laboratory (Bar Harbor, ME). Mice were housed in an AAALAC (Association for Assessment and Accreditation of Laboratory Animal Care) accredited animal facility, which was pathogen-free, humidity/temperature controlled and supplied food and water ad libitum. All animal procedures were approved by the NC State University Institutional Animal Care and Committee (IACUC). Mice were housed 3 per cage according to their respective treatment groups and genotypes– vehicle control (WT), vehicle control (*Par2* KO), MWCNTs (WT), MWCNTs (*Par2* KO), HDM extract (WT), HDM extract (*Par2* KO), MWCNTs + HDM extract (WT), and MWCNTs + HDM extract (*Par2* KO).

### Exposure of mice to MWCNTs and HDM extract

For all exposure procedures, which consisted of three exposure sessions in both the sensitization phase (days 1, 3, 5) and the challenge phase (days 15, 17, 19), mice were exposed by oropharyngeal aspiration (OPA) to 50 µL of the following treatments: Vehicle solution control, MWCNTs, HDM extract, or both. All treatments were prepared in vehicle solution which consisted of 0.1% Pluronic F-68 non-ionic surfactant (ThermoFisher Scientific) in DPBS (Sigma) and were vortexed immediately before delivery to mice by  oropharyngeal aspiration (OPA) under isoflurane anesthesia. OPA is a well-established surrogate method to inhalation for pulmonary delivery of particles and other agents into the lungs of mice [25–29]. For the initial experiment using MWCNTs and a relatively high dose of HDM extract, male wildtype (WT) C57BL/6J mice were exposed to vehicle or 10 µg/mouse of HDM per dosing session (0.4 mg/kg body weight) with or without 12.5 µg of MWCNTs (0.5 mg/kg). In this experiment, the cumulative dose of HDM extract was 2.4 mg/kg and the cumulative dose of MWCNTs was 3 mg/kg. In a separate experiment that utilized relatively low doses of HDM extract, male WT C57BL/6J mice were exposed to 0 µg, 0.1 µg, 0.5 µg, or 1 µg/mouse of HDM extract per dosing session with or without 12.5 µg of MWCNTs. For the experiment comparing WT and *Par2* KO mice, male WT C57BL/6J mice and male *Par2* KO mice were exposed to vehicle or 0.5 µg/mouse HDM extract (0.02 mg/kg) per dosing session with or without 12.5 µg of MWCNTs per dosing session.

### Necropsy and sample collection

Necropsy was performed on day 22 to observe pro-inflammatory and pro-fibrotic responses in the lungs of mice. Mice were euthanized with an intraperitoneal injection of pentobarbital. Bronchoalveolar lavage fluid (BALF) was collected from each mouse by cannulating the trachea and conducting lavages of the lungs with 0.5 ml of chilled Dulbecco's phosphate-buffered saline (DPBS) two times. The BALF was centrifuged at 140 xg for 5 min and the resulting supernatant was transferred to a separate set of tubes and utilized for analysis of protein, LDH, and cytokines/chemokines. The cell pellet was resuspended in 500 μL DPBS and used to analyze inflammatory cells. For histopathology, the left lung lobe was fixed in neutral buffered formalin (VWR, Radnor, PA) for 24 h, then transferred to 70% ethanol for 3 days before being embedded in paraffin. For mRNA analysis, a right superior lung lobe was stored in RNAlater (Fisher Scientific, Waltham, MA). For protein analysis, the right medial and inferior lung lobes were snap frozen in liquid nitrogen and stored at − 80 °C.

### BALF inflammatory cell counts

Total BALF cell counts were performed using a hemocytometer. For differential cell counts, 100 µl of BALF was centrifuged onto glass slides using a Cytospin 4 centrifuge (ThermoFisher, Waltham, MA) and the slides were then fixed and stained with the Diff-Quik stain set (Siemens, Newark, DE). Cell differentials were quantified by counting 500 cells per slide using an Olympus light microscope BX41 (Center Valley, PA) to determine relative numbers of macrophages, neutrophils, eosinophils, and lymphocytes.

### Cytokine analysis in BALF

DuoSet enzyme-linked immunosorbent assay (ELISA) kits (R&D Systems, Minneapolis, MN) were used according to the manufacturer’s protocol to quantify protein levels of cytokines C-X-C motif chemokine ligand 1 (CXCL1), C-C motif chemokine ligand 2 (CCL2), interleukin 1 beta (IL-1β), interleukin 6 (IL-6), osteopontin (OPN), and tumor necrosis factor alpha (TNF-α) from BALF. Cytokine concentrations were derived from the absorbance values measured at 450 nm with a background correction at 540 nm using the Multiskan EX microplate spectrophotometer (ThermoFisher, Waltham, MA). GraphPad Prism 9 software was used to generate standard curves based on the manufacturer’s protocol, and concentration values were interpolated.

### Cytotoxicity and total protein in BALF

Lactate dehydrogenase (LDH) activity in BALF was assayed as an indicator for pulmonary cytotoxicity with the “Pierce LDH Cytotoxicity Assay Kit” (ThermoFisher, Waltham, MA), according to the manufacturer’s instructions. Absorbance values were measured at 450 nm using a Multiskan EX microplate spectrophotometer (ThermoFisher). Total protein concentration in BALF was determined, according to the manufacturer’s protocol with the “Pierce BCA Protein Assay Kit” (ThermoFisher).

### qRT-PCR

Applied Biosystems high-capacity cDNA reverse transcription kit (ThermoFisher Scientific, Waltham, MA) was used to create cDNA from the mRNA isolated from the right lung lobes using Quick-RNA™ MiniPrep (Zymo Research, Irvine, CA) according to the manufacturer’s instructions. The FastStart Universal Probe Master (Rox) (Roche, Basel, Switzerland) was then used to run Taqman qPCR on the Applied Biosystems QuantStudio3 Real-Time PCR System Thermal Cycling Block (ABI, Foster City, CA) to determine the comparative C_T_ (ΔΔC_T_) fold change expression of specific mRNAs (*Ccl-11*, *Tgf-β1*, *Col1a1*, *Col1a2*, *Stat6*) normalized to *β-actin* as the endogenous control.

### Immunoblotting

Whole lung lysate was prepared from snap-frozen right lung lobe. Frozen lung samples were transferred to 1.5 mL centrifugation tubes containing lysis buffer (20 mM Tris–HCl, 150 mM NaCl, 1 mM EDTA, 1 mM EGTA, 1% Triton X-100, 1 mM Na_3_VO_4_, 1 × Halt™ Protease Inhibitor Cocktail, in DPBS). One 35 mm stainless steel bead was inserted into each 1.5 mL centrifugation tube containing lung sample and lysis buffer to facilitate tissue homogenization. Mini Bead Mill Homogenizer (VWR International) was used with a speed setting of 4 for 1 min. The resulting mixture was spun down at 11,100 xg for 4 min. The protein concentration of the supernatant was determined using the Pierce BCA Protein Assay Kit (ThermoFisher Scientific, Waltham, MA). Absorbance was read at 450 nm with a correction at 540 nm using the Multiskan EX microplate spectrophotometer (ThermoFisher, Waltham, MA). Samples were loaded onto a Novex™ 4–12% SDS-PAGE gel (Invitrogen, Carlsbad, CA), and separated by electrophoresis and transferred onto PVDF membranes. Membranes were blocked for one hour and incubated overnight in 1:1000 dilution of rabbit or mouse primary antibodies purchased from Cell Signaling Technology (phosphorylated STAT6 at Tyr640, #56554S; STAT6, #5397S; phosphorylated NF-κB at Ser536, #3033S; NF-κB, #8242S; Arginase-1, #93668S; phosphorylated smad2 at Ser465/467 and smad3 at Ser 423/425, #8828S; Smad 2/3, #5678S; and β-actin, #4967L). Following primary antibody incubation, the membranes were washed and incubated in 1:2500 dilution (Cell Signaling Technology, Danvers, MA) with horseradish peroxidase-conjugated secondary anti-rabbit antibody. Enhanced chemiluminescence (ECL) Prime Western Blotting Detection Reagent (Cytiva, Marlborough, MA) was used in accordance with manufacturer instructions to facilitate HRP-induced chemiluminescence, and resulting signals were captured using Amersham Imager 680 (GE Life Sciences, Marlborough, MA). Semi-quantitative densitometry was performed using ImageQuant software (GE Life Sciences, Marlborough, MA).

### Histopathology and quantitative morphometry of airway fibrosis and mucous cell metaplasia

The left lung was cut into three cross sections, which were embedded in paraffin, and 5 micron histologic sections were mounted on charged glass slides. Sections were stained with the following: Hematoxylin and eosin (H&E) to assess pro-inflammatory tissue reactions, Gomori’s trichrome for collagen deposition, and Alcian blue/periodic acid-Schiff (AB/PAS) for mucus production. Airway fibrosis, based on Gomori’s trichrome-stained slides, was assessed by measuring thickness of the collagen layer, surrounding the airways, using an area/perimeter ratio method, as described previously [30, 31]. Approximately 10 airways per lung cross-sections per mouse (3 cross-sections per mouse, resulting in a total of 30 photomicrographs per mouse) that fit to our criteria (circular airways that fit in the field of view) were photographed at 100× magnification using an Olympus BX41 light microscope (Center Valley, PA). To determine the area/perimeter ratio, round to oval shaped airways under 500 × 500 μm (H × W) were imaged at 100× . The lasso tool in Adobe Photoshop CS5 was used to surround trichrome positive collagen around the airways, giving the outer area, and to surround the basement membrane, giving the inner area and airway circumference (perimeter). The difference between the outer and inner area was divided by the circumference giving the area/perimeter ratio. All measurements were performed in a blinded manner. Mucous cell metaplasia and airway mucus production were assessed by imaging all airways under approximately 500 × 500 μm (H × W) in each AB/PAS-stained sample and quantifying the area of positive staining in ImageJ (National Institutes of Health) as a percent area.

### Immunofluorescence analysis of arginase-1 (ARG-1) in lung tissue

Sequential dual immunofluorescence (IF) was performed on paraffin-embedded tissues sectioned at 5 microns. Tissue sections were labeled for the following antigens: F4/80 (70,076, Cell Signaling Technology) and Arginase 1 (ARG-1) (93,668, Cell Signaling Technology). This assay was carried out on the Bond III fully automated slide staining system (Leica Biosystems) using the Bond Research Detection kit (DS9455). Slides were dewaxed in Bond Dewax solution (AR9222) and hydrated in Bond Wash solution (AR9590). Heat induced antigen retrieval was performed at 100ºC in Bond-Epitope Retrieval solution 1 pH-6.0 (AR9961) for 20 min. After pretreatment, tissues were blocked, and primary antibodies were diluted as follows: F4/80 at 1:2000 and Arginase 1 at 1:200. Ready-to-use secondary antibody, Leica’s Novolink Polymer (RE7260-CE) was used followed by either TSA Cy5 (SAT705A001EA, Akoya Biosciences) or TSA Cy3 (SAT704A001EA, Akoya Biosciences) to visualize the target of interest. Nuclei were stained with Hoechst 33,258 (Invitrogen). The stained slides were mounted with ProLong Gold antifade reagent (P36930, Life Technologies). Positive controls were included for each assay. The slides were imaged using the 20X objective on a Leica Versa whole slide scanner (Leica Biosystems). A board-certified veterinary pathologist completed the analyses performed by the UNC Pathology Services Core using Definiens Architect XD 64 2.7.0 software (Cambridge, MA). Lung sections were detected using the automatic detection mode with a minimum tissue size of 310,000 µm^2^ minimum. Cell nuclei stained with DAPI were identified using a threshold of 0.45 and a typical nuclear size of 68.1 µm^2^. Partial nuclei or nuclei outside the plane of view smaller than 35 µm^2^ were excluded from the analysis. The expression of F4/80 and ARG-1 were determined using thresholds of 2500 and 4000, respectively. The computer also detected F4/80 and ARG-1 cellular co-expression, relative staining intensity, and cellular density. The Definiens output was validated by a veterinary pathologist quantitating positive nuclei in randomly selected groups of 100 nuclei.

To enhance rigor and reproducibility, the same multiplexed fluorescence images from tissue sections were analyzed with QuPath 0.4.3 [32]. The annotation area for each lung tissue section was selected manually to provide an estimated area of the tissue sections per mice in µm^2^. The expression of DAPI, ARG-1, and F4/80 were determined using detection thresholds ranging from 5000 to 7500 based on fluorescence intensity. Nuclei with less than 10 µm^2^ or greater than 400 µm^2^ were excluded. The software also detected DAPI and ARG-1 co-expressing and ARG-1 and F4/80 co-expressing cells using the aforementioned detection thresholds.

### Statistical analysis

One-way ANOVA with Tukey’s post hoc test or Student’s t-test was utilized to evaluate differences between treatment groups (GraphPad Prism, version 9.0, La Jolla, CA). Two-way ANOVA with a Tukey’s post hoc test was utilized to evaluate differences among treatment and genotypic groups. All data represent the mean ± SEM of three to six animal replicates.

## Results

### Pulmonary exposure to high dose HDM extract via oropharyngeal aspiration (OPA) increases lung inflammation and results in eosinophilic infiltration

Using the sensitization and challenge protocol illustrated in Fig. 1B, male C57BL/6 mice were exposed by oropharyngeal aspiration (OPA) a total of 6 times over a period of 3 weeks to the following treatments in 50 µL vehicle each dosing session: 0.1% pluronic DPBS solution as the vehicle control, 12.5 µg of MWCNTs (0.5 mg/kg), 10 µg of HDM extract (0.4 mg/kg), or combination of MWCNTs and HDM extract. In this experiment, the cumulative dose of HDM extract was 2.4 mg/kg and the cumulative dose of MWCNTs was 3 mg/kg. In this initial attempt to explore the consequences of HDM extract and MWCNT co-exposure via OPA, it was found that MWCNTs alone did not produce a significant increase in the number of cells present in the BALF. In contrast, exposure to a relatively high dose of HDM extract resulted in a robust increase in total number of BALF cells present. Co-exposure to MWCNTs and high dose HDM extract resulted in similar total BALF cell counts as what was observed with BALF samples from mice exposed to high dose HDM extract alone (Fig. 1C). Lung sections stained with hematoxylin and eosin (H&E) showed increased inflammation in the lungs of mice exposed to either high dose HDM extract or the combination of MWCNTs and high dose HDM extract, but not in the lungs of mice exposed to MWCNTs alone (Fig. 1D). Additionally, high magnification oil immersion light microscopy of lung tissue slides from mice co-exposed to MWCNTs and high dose HDM extract showed that MWCNTs were present within eosinophilic granulomatous lesions found near airways (Additional file 2: Fig. S1).

### Co-exposure to MWCNTs synergistically enhances lung inflammation in the presence of relatively low doses of HDM extract

Next, a separate experiment was conducted on male C57BL/6 mice using lower doses of HDM extract consisting of 0.1 µg (0.004 mg/kg), 0.5 µg (0.02 mg/kg), and 1 µg (0.04 mg/kg) of HDM extract per dosing, which were 100-fold, 20-fold, and tenfold, respectively, lower than the 10 µg of HDM extract per dosing used in the initial experiment. These relatively low doses of HDM extract alone did not produce significant lung inflammation. However, co-exposure to MWCNTs in the presence of all three low doses of HDM extract significantly increased the number of cells present in the BALF compared to MWCNTs alone or low dose HDM extract alone (Fig. 1E). Qualitative assessment of H&E-stained lung sections from mice indicated a similar pattern of increased inflammation only in the MWCNT + HDM co-exposed group and not in groups exposed to either MWCNTs or HDM extract alone (Fig. 1F). These data clearly demonstrated that MWCNTs synergistically increased lung inflammation induced by relatively low doses of HDM extract, that alone produced little if any pro-inflammatory effect. All subsequent experiments with wildtype (C57BL/6) mice and *Par*2 KO utilized a dose of 0.5 µg HDM extract (0.02 mg/kg body weight).

### PAR2 does not mediate the exacerbation of HDM-induced lung inflammation by MWCNTs

Male wildtype (WT) and *Par2* KO mice (JAX Stock #004993; The Jackson Laboratory, Bar Harbor, Maine) were exposed to 50 µL of 0.1% Pluronic DPBS solution as the vehicle control, 12.5 µg of MWCNTs (0.5 mg/kg), 0.5 µg of HDM extract (0.02 mg/kg), or combination of MWCNTs and HDM per dosing session via OPA according to the dosing strategy shown in Fig. 2A. Genotyping confirmed the identity of WT and *Par2* KO mice used in this study (Additional file 3: Fig. S2). Cytospins of BALF from the lungs of mice showed that MWCNT and HDM extract co-exposure produced a marked increase in eosinophils compared to mice exposed to either MWCNTs or HDM extract alone (Fig. 2B). Quantification of BALF cell counts showed MWCNTs co-exposed with HDM extract synergistically increased numbers of total cells in BALF that were similar between WT and *Par2* KO (Fig. 2C). Differential cell counting of BALF cells confirmed that only the mice co-exposed with MWCNT and HDM extract displayed an increased number of eosinophils in the BALF (Fig. 2D). Interestingly, MWCNT exposure alone increased the number of neutrophils in BALF in both genotypes (Fig. 2E). Taken together, it was evident that co-exposure with MWCNTs and HDM extract suppressed the MWCNT-induced neutrophilia (Fig. 2E). The relative number of macrophages per 500 BALF cells counted were reduced in both genotypes (Fig. 2F). The lack of any difference in total or differential cell counts in BALF among genotypes suggested that PAR2 did not play any significant role in inflammatory cell recruitment after exposure to HDM extract and MWCNTs. Like cell counts, LDH and total protein in BALF were synergistically increased by MWCNT and HDM co-exposure with no observable differences between WT and *Par2* KO mice (Fig. 2G and H). Protein levels of CXCL-1 and CCL2 in BALF detected by ELISA were significantly increased by co-exposure to HDM extract and MWCNTs, with higher levels in par2 KO mice compared to WT mice (Additional file 4: Fig. S3). No changes were seen among treatment groups or genotypes for IL-1β, IL-6, OPN or TNF-α (data not shown).

### PAR2 contributes to the exacerbation of airway fibrosis induced by co-exposure to MWCNTs and HDM extract

**Fig. 3:**
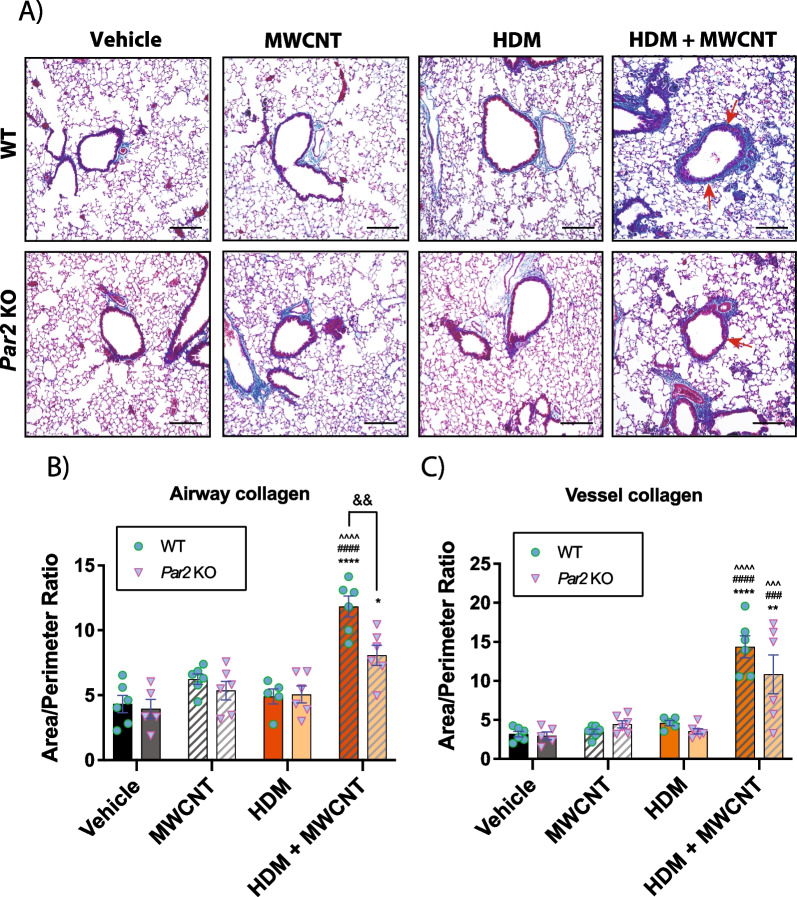
Airway and vessel fibrosis in the lungs of WT and *par2* KO mice exposed to HDM and MWCNTs. **A** Representative images of trichrome stained lung sections. **B** Quantification of area to perimeter ratio around airways of Gomori’s trichome stained lung sections. **C** Quantification of area to perimeter ratio around major blood vessels of Gomori’s trichome stained lung sections. n = 6. ****p < 0.0001, ***p < 0.001 when compared to respective vehicle control groups, ^^^^*p* < 0.0001, ^^^*p* < 0.001 when compared to respective HDM groups, ####*p* < 0.0001, ##*p* < 0.01 when compared to respective MWCNT groups, &&*p* < 0.01 when compared to genotypical counterpart as determined by two-way ANOVA with Tukey’s post hoc analysis. Magnification bars in each panel = 100 µm

Histopathological evaluation of fibrosis in the lungs of exposed mice was conducted using Gomori’s trichrome staining. While no discernable differences were observed from groups exposed to either MWCNTs or HDM extract alone, co-exposure to MWCNTs and HDM extract significantly increased trichrome-positive collagen around airways and pulmonary blood vessels in the lungs of mice, and this was more prominent in WT mice (Fig. 3A). Quantitative morphometry of trichrome-positive lesions assessed by a previously established area/perimeter ratio method [30, 31] revealed a significant difference in airway fibrosis in WT mice co-exposed to both MWCNTs and HDM extract when compared to that of *Par2* KO counterparts (Fig. 3B). Fibrosis around blood vessels displayed a similar trend but the difference of the means between the two genotypes did not reach statistical significance (Fig. 3C). Additionally, AB-PAS staining was performed on lung tissues to determine mucous cell metaplasia in the lungs of mice. Mucous cell metaplasia was synergistically increased by co-exposure to MWCNTs and HDM, but quantitative morphometry demonstrated no significant difference between WT and *Par2* KO mice (Additional file 5: Fig. S4). Quantitative morphometry was also performed to assess the size and number of granulomas in the lungs of WT and *Par2* KO mice. No significant differences between genotypes were observed for either granuloma number or size induced by exposure to MWCNTs or the combination of MWCNTs and HDM extract (Additional file 6: Fig. S5).

### PAR2 differentially regulates key signaling components of the innate immune response and fibrosis

**Fig. 4:**
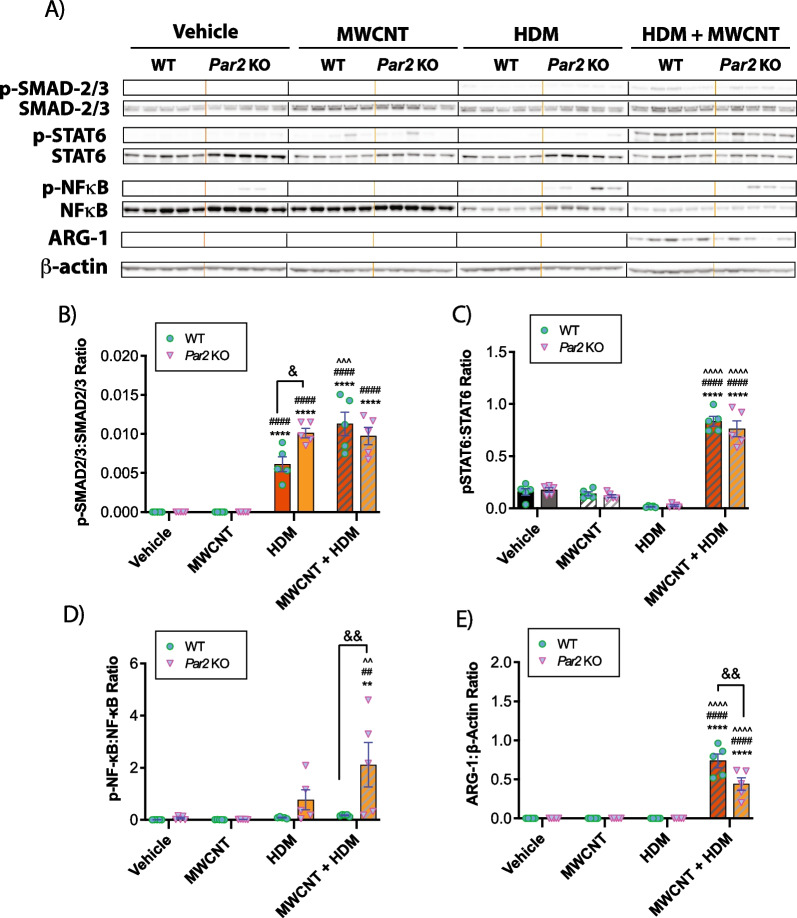
Western blotting of cell signaling mediators in the lungs of WT and *Par*2 KO mice exposed to HDM extract and MWCNTs. **A** Western blots of whole lung lysates from all animals evaluated (see Additional file 6: Fig. S5 for the original uncropped western blots). **B** Densitometry of p-SMAD2/3 to total SMAD2/3. **C** Densitometry of p-STAT6 normalized against total STAT6. **D** Densitometry of p-NF-κB normalized against total NF-κB. **E** Densitometry of ARG-1 against β-Actin. n = 5. ****p < 0.0001, **p < 0.01 when compared to respective vehicle control groups, ^^^^*p* < 0.0001, ^^*p* < 0.01 when compared to respective HDM groups, ####*p* < 0.0001, ##*p* < 0.01 when compared to respective MWCNT groups, &&*p* < 0.01, &*p* < 0.05 between genotypes as determined by two-way ANOVA with Tukey’s post hoc analysis

Whole lung lysates were evaluated by western blot analysis to investigate specific molecular mechanisms that could contribute to the exacerbation of the lung inflammatory or fibrotic response to MWCNTs and HDM extract. Western blots from all animals evaluated are shown in Fig. 4A, while the original uncropped western blot data are shown in Additional file 7: Fig. S6. Semi-quantitative densitometry from all animals is shown in Fig. 4B–E. Co-exposure to HDM extract and MWCNTs increased the phosphorylation of SMAD2/3, but there were no significant differences between genotypes (Fig. 4B). Exposure to HDM extract alone showed increased Smad2/3 phosphorylation *Par2* KO mice compared to WT mice (Fig. 4B). Phosphorylated (p)-STAT6 was induced approximately fivefold by MWCNT + HDM extract co-exposure similarly in both genotypes, while levels of total STAT6 remained relatively constant among treatments and genotypes (Fig. 4C). Phosphorylation of NF-κB was increased in lung lysates from the majority of *Par2* KO mice treated with HDM extract or co-exposed to HDM extract and MWCNTs (Fig. 4D). Densitometric analysis of each whole lung lysate sample revealed that NF-κB activation was significantly increased by HDM extract alone and further increased by co-exposure to MWCNTs and HDM extract in *Par2* KO mice, but not in WT mice (Fig. 4D). Arginase-1 (ARG-1) was expressed only in the MWCNT + HDM co-exposure group and was significantly reduced in *Par2* KO mice compared to WT mice (Fig. 4E).

### Co-exposure to MWCNTs and HDM extract synergistically increases Arg-1, Col1a1 and Ccl-11 (eotaxin) gene expression

**Fig. 5:**
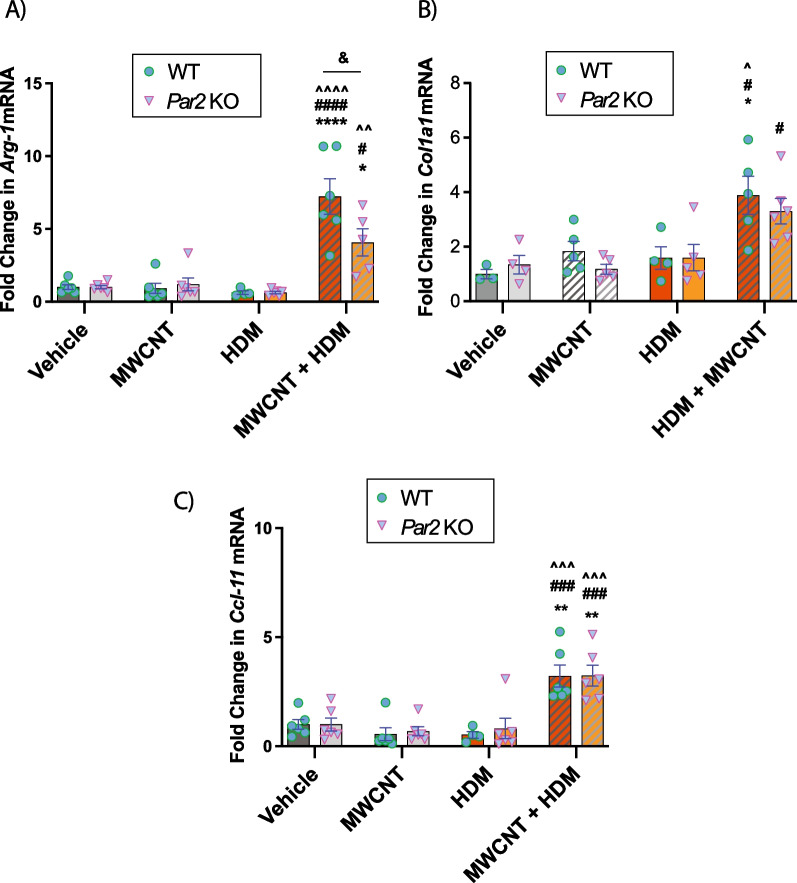
Lung mRNAs of mediators of eosinophilia and fibrosis in the lungs of WT and PAR2 KO mice exposed to HDM extract and MWCNTs. **A** Relative *Arg-1* mRNA expression. **B** Relative *Col1a1* mRNA expression. **C** Relative *Ccl-11* mRNA expression. n > 4. ****p < 0.0001, **p < 0.01, *p < 0.05 when compared to respective vehicle control groups, ^^^^*p < *0.0001, ^^^*p* < 0.001, ^^*p* < 0.01, ^*p* < 0.05 when compared to respective HDM groups, ####*p* < 0.0001, ###*p* < 0.001, #*p* < 0.05 when compared to respective MWCNT groups, &*p* < 0.05 between genotypes as determined by two-way ANOVA with Tukey’s post hoc analysis

Gene expression analysis via qRT-PCR revealed significantly increased *Arg-1* expression in MWCNTs and HDM extract co-exposed groups for both genotypes (Fig. 5A). Moreover, *Arg-1* mRNA expression induced by co-exposure to HDM extract and MWCNTs was significantly reduced in *par2* KO mice compared to WT mice (Fig. 5A), correlating to protein expression of ARG-1 shown in Fig. 4. Total lung *Col1a1* mRNA was significantly higher after co-exposure to HDM extract and MWCNTs compared to HDM extract or MWCNTs alone in either WT or *Par2* KO mice (Fig. 5B). However, there was no significant difference in *Col1a1* between genotypes. The mRNA expression of *Ccl-11*, a primary eosinophil chemokine, was also found to be significantly increased in the MWCNT and HDM extract co-exposure group but was not different between genotypes (Fig. 5C). The pattern of *Ccl-11* corresponded to BALF cellularity analysis which showed eosinophilic recruitment in MWCNT and HDM extract co-exposed groups (Fig. 2). The mRNA expression of *Tgf-β1*, *Col1a2* and *Stat6* was not significantly changed among treatment groups or genotypes (data not shown).

### Mononuclear phagocytes are a major source of ARG-1 expression

**Fig. 6:**
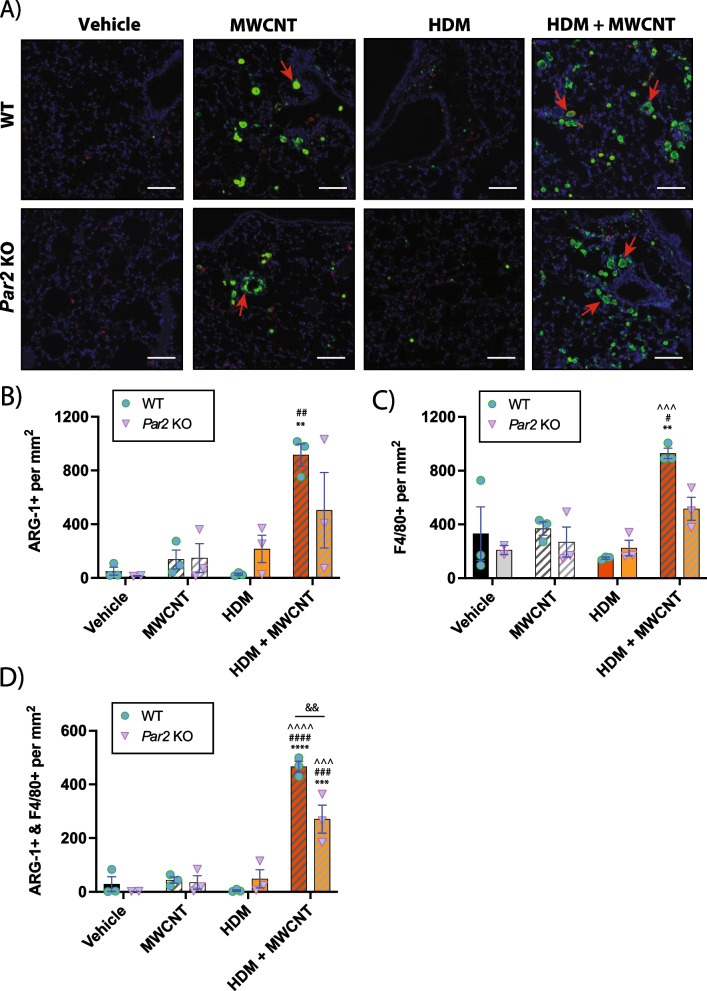
Immunohistochemistry for localization of ARG-1 and F4/80 in tissue sections from WT and *Par2* KO mice exposed to MWCNTs, HDM extract or MWCNTs and HDM extract. **A** Representative microscopic images of lung tissue showing ARG-1 + cells (green), F4/80 + cells (red), and DAPI + cells (blue). Red arrows indicate ARG-1 + mononuclear cells associated with MWCNTs. White bars = 50 µm. **B** Semi-quantitative analysis derived from Definiens software showing the numbers of ARG-1 + cells per mm^2^ lung tissue sections per animal. **C** Numbers of F4/80 + cells per mm^2^ lung tissue per animal analzed by Definiens software. **D** Numbers of ARG-1 + and F4/80 + cells per mm^2^ lung tissue per animal analzed by Definiens software. The data represent 3 animals per group for each genotype

To characterize the abundance and localization of ARG-1 protein expression, lung tissue sections were analyzed via immunohistochemistry using fluorescence probes for ARG-1 and F4/80, a monocyte/macrophage marker (Fig. 6A). The overall abundance of ARG-1 expression was assessed by semi-quantitative analysis using Definiens software on three animals per group and revealed a synergistic increase in the MWCNT and HDM extract co-exposure group for both genotypes (Fig. 6B). The percentage of F4/80 + cells was significantly also higher in the MWCNT and HDM extract co-exposure group for both WT and *Par2* KO groups (Fig. 6C). Cells that were both ARG-1 + and F4/80 + after exposure to HDM extract and MWCNTs were significantly different between genotypes (Fig. 6D). The F4/80 marker showed that macrophages were a source of the ARG-1 expression. An independent semi-quantitative analysis using QuPath software yielded similar results (Additional file 8: Fig. S7). Upon qualitative analysis, it was evident that most of the ARG-1 + cells were round and were mononuclear with some containing MWCNT inclusions in the cytoplasm, indicating that these cells are mononuclear phagocytes.

## Discussion

Elucidating the mechanisms through which inhaled nanoparticles exacerbate allergen-induced lung disease in experimental animals remains an important area of research to better understand how inhaled particles exacerbate asthma in humans. For example, particulate matter (PM)-mediated oxidative stress has previously been demonstrated to enhance an immune response to allergens in rodent models [33, 34]. Moreover, diesel exhaust particle (DEP) exposure significantly increased allergen specific Th2 and Th17 cells leading to airway hyperresponsiveness [35]. Additionally, co-exposure to cigarette smoke strongly upregulated allergen induced Th2 responses [36]. Regarding ENMs specifically, we previously reported that MWCNTs, in the presence of HDM extract, elevated allergen-induced responses by increasing serum IgE levels, airway fibrosis, and mucous cell metaplasia [30]. Others have reported that MWCNTs increase ovalbumin (OVA)-induced serum IgE, numbers of eosinophils in BALF, and production of Th2 cytokines in the lungs of mice [12]. In the current study, we clearly demonstrated an exacerbated pulmonary immune response in mice upon co-exposure to MWCNTs and HDM extract which was characterized by a synergistic increase in eosinophilic inflammation in addition to increased phosphorylation of STAT-6, and increased expression of ARG-1 protein and *Arg-1*, *Cc11* and *Col1a1 mRNAs*.

We also evaluated the potential role of PAR2 in MWCNT-driven exacerbation of the pulmonary allergic response to HDM extract using *Par2* KO mice. PAR2 is a receptor highly expressed in the lung and is important in airway remodeling [37, 38]. Previous studies have demonstrated PAR2-mediated fibrosis of various organs, including the lung, supported by evidence of increased TGF-β production, activation of epidermal growth factor receptor, increased expression of pro-fibrotic genes, and myofibroblast differentiation [39–44]. Using doses of MWCNTs and HDM extract that, alone, displayed very low levels of immunological or inflammatory responses in the lung, we demonstrated a strong adjuvant-like effect following repeated co-exposure to both MWCNTs and HDM extract. The strong adjuvant-like effect of MWCNTs in the presence of HDM extract produced an allergic inflammatory response that was almost entirely eosinophilic. We previously reported that eosinophilic lung inflammation induced by HDM extract and MWCNTs was abolished in *Stat6* KO mice [30]. However, eosinophilic lung inflammation induced by the combination of MWCNTs and HDM extract in the present study was not different between WT and *Par2* KO mice and no genotypic difference in STAT6 activation was observed. This suggested that PAR2 may not be directly involved in allergic lung inflammation exacerbated by MWCNTs and that PAR2 does not seem to directly impact STAT6-mediated eosinophilic lung inflammation.

Despite the absence of any difference in eosinophilic lung inflammation or changes in LDH and total protein in the BALF from WT versus *par2* KO mice, we did find evidence that PAR2 mediates airway fibrosis. Morphometric analysis of airways in lung tissue sections of mice exposed to both MWCNTs and HDM extract revealed significantly reduced airway collagen in the lungs of *Par2* KO mice compared to WT mice. Previous animal studies involving bleomycin-induced pulmonary fibrosis mouse models showed that the absence or inhibition of PAR2 was associated with a significant reduction in various pro-fibrotic measurements, including a reduction in total lung collagen, reduction in epithelial-mesenchymal transition, reduction in severity and extent of fibrotic lesions, and reduction in pro-fibrotic signaling molecules such as TGF-β and monocyte chemotactic protein 1 (MCP-1), also known as CCL2 [22, 45, 46]. However, while we observed significantly higher phosphorylation levels of SMAD2/3 protein (Fig. 4) in the lungs of mice co-exposed to MWCNTs and HDM extract, we did not observe any statistical difference in p-SMAD2/3 between genotypes as assessed by western blot analysis. Likewise, *Col1a1* mRNA levels were significantly higher in the lungs of both WT and *Par2* KO mice co-exposed to HDM extract and MWCNTs (Fig. 5). Collectively, the data suggested that while the increase in collagen deposition observed in both genotypes may be dependent on TGF-β1 signaling, the reduced airway collagen specific to the lungs of *Par2* KO under the parameters of this study was likely independent of TGF-β1 signaling.

In our efforts to elucidate potential mechanisms responsible for the observed reduction in airway collagen levels in *Par2* KO mice co-exposed to both MWCNT and HDM extract, we found significantly reduced mRNA and protein levels for the pro-fibrotic mediator arginase-1 (ARG-1) in lungs from *Par2* KO mice co-exposed to MWCNTs and HDM extract. Dual immunohistochemistry for ARG-1 and F4/80 revealed that ARG-1 was expressed in mononuclear phagocytes in the lungs of both WT and *Par2* KO mice, and many ARG-1 + phagocytes contained MWCNTs. Semi-quantitative morphometry revealed that ARG-1 + cells that were also F4/80 + were significantly reduced in *Par2* KO mice compared to WT mice. Moreover, numbers of F4/80 + cells were reduced in *Par2* KO mice, suggesting that PAR2 may play a role in regulating macrophage recruitment and/or polarization to ARG-1-expressing M2 macrophages. It is important to note that some ARG-1 + cells were not F4/80 + , indicating that macrophages are not the only source of ARG-1 in the lung. In addition to macrophages, lung epithelial cells and fibroblasts have been reported to express PAR2. For example, a comparative study of human lung sections from healthy donors and donors with idiopathic pulmonary fibrosis (IPF) showed that healthy donors expressed PAR2 in alveolar macrophages and alveolar type 2 cells, while those of the IPF donors expressed PAR2 in hyperplastic alveolar type 2 cells and fibroblasts [37].

**Fig. 7:**
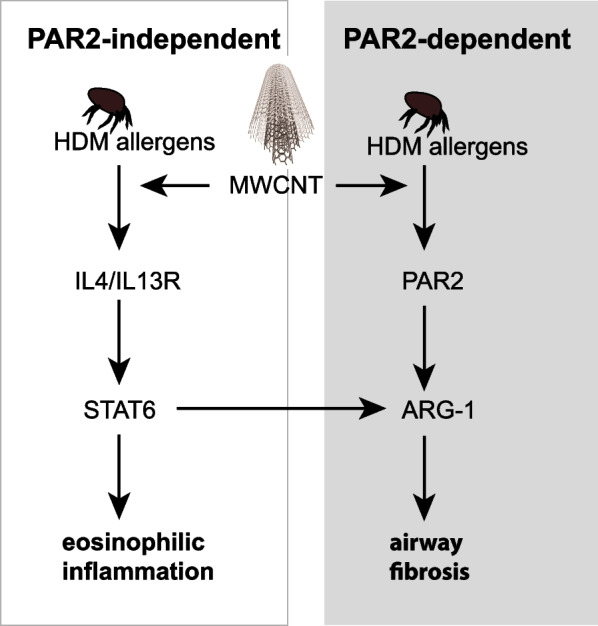
Hypothetical illustration showing PAR2-independent and PAR2-dependent components of allergic lung disease in mice caused by co-exposure to MWCNTs and HDM allergens. MWCNTs enhance HDM allergen-induced eosinophilic inflammation through a STAT6-dependent mechanism that does not involve PAR2. However, PAR2 is postulated to promote airway fibrogenesis by increasing the expression of ARG-1

One of the contributing factors to the progression of collagen deposition and fibrosis is the alteration of cellular metabolism to favor the production and activity of specific components, such as amino acids, instrumental in collagen synthesis and deposition. In this context, ARG-1 metabolizes l-arginine into l-ornithine, which is then further metabolized to l-proline, the second most prevalent amino acid in collagen [47]. Therefore ARG-1 expression is often considered a key marker of fibrosis [48–50]. Bleomycin-induced pulmonary fibrosis in mice revealed significant upregulation of ARG-1 [48]. Conversely, the down-regulation of ARG-1 in mice attenuated pulmonary fibrosis [49]. Therefore, in concurrence with the previously documented roles of ARG-1 in pulmonary fibrosis, our data showing reduced ARG-1 expression in the lungs of *Par2* KO mice not only further strengthens the notion that ARG-1 expression is a contributing factor to reduced airway collagen deposition but also introduces potential interplay between PAR2 and ARG-1. Additionally, our findings re-emphasize previous arguments on the involvement of cellular metabolic plasticity as a contributing factor in processes necessary for fibrogenesis [51–53]. While ARG-1 expression is instrumental in shifting fibroblast/myofibroblast amino acid metabolism that ultimately activates intracellular machinery to favor the production and deposition of collagen, ARG-1 has also been implicated in lung immune functions as it is widely considered to be one of the classical biomarkers of alternatively activated macrophages (M2) [54]. The fact that both activation of fibroblasts and myofibroblasts as well as alternative activation of macrophages have long been accepted as canonical events in pulmonary fibrogenesis further highlights the importance of our observation of reduced airway fibrosis and ARG-1 expression in lungs of *Par2* KO mice following co-exposure to MWCNTs and HDM extract. As illustrated in Fig. 7, our findings demonstrate that PAR2 promotes ARG-1 mRNA and protein expression. However, further work is necessary to determine whether ARG-1 is responsible for airway fibrosis caused by co-exposure to MWCNTs and HDM extract in the present study.

Others have previously reported that PAR2 may be critical in the airway response to allergens. For example, the airways of *Par2* KO mice exhibited diminished eosinophil infiltration 24 h following ovalbumin (OVA) sensitization and challenge [55]. Both deficiency and inhibition of PAR2 induced in experimental models have previously resulted in significantly decreased numbers of eosinophils in the lung as well as decreased eotaxin protein levels upon allergen challenge [24, 56]. Furthermore, *Par2* KO mice showed a reduced influx of eosinophils and reduced protein leakage in the bronchoalveolar space compared to similarly exposed WT mice following repeated airway exposure to HDM allergens [23]. However, we did not observe any statistically significant difference between the genotypes in eosinophil infiltration into the lung (Fig. 2) or the expression of *Ccl-11* mRNA encoding lung eotaxin (Fig. 5) following exposure of mice to HDM extract and MWCNTs. One potential explanation for the lack of difference in genotypes is the temporal aspect of our study compared to other studies. Schmidlin et al. reported that the extent of the cellular infiltrate into BALF following repeated allergen challenge was significantly reduced in *Par2* KO mice at 24 h post exposure but not at 72 h post exposure [55]. Similarly, mice overexpressing PAR2 that underwent similar treatment exhibited a significant increase in cellular infiltrate into BALF at 24 h post-exposure but not at 72 h post-exposure [57]. Our current study implemented a repeated-exposure design over 22 days but conducted sample collection 72 h following the last allergen challenge. This could indicate that the role of PAR2 in mediating inflammation may be limited to early phases of inflammation and may have less influence over time during prolonged inflammation.

The exact function of PAR2 as a mediator of the pro-inflammatory response is still unclear due to conflicting reports that implicate PAR2 in both pro-inflammatory and anti-inflammatory roles [58–60]. As articulated in detail by Zhuo et al. in their recent review article on PARs, the context in which these receptors are stimulated may have a decisive role on what role PARs may play in inflammation [61]. While the current study did not demonstrate reduced eosinophilic lung inflammation in *Par2* KO mice, we observed increased phosphorylation of NF-κB protein in whole lung lysates from *Par2* KO mice exposed to HDM extract or co-exposed to HDM extract and MWCNTs, indicating that PAR2 regulates some aspects of inflammation. Moreover, BALF cytokine analysis revealed that CXCL-1 cytokine levels from mice co-exposed to MWCNTs with HDM extract also displayed similar genotypic differences as NF-κB activation, with *Par2* KO mice expressing a greater amount of the neutrophil chemokine CXCL-1. We did not, however, observe a difference in corresponding neutrophil counts between WT and *Par2* KO mice in our BALF cellularity analysis. Nevertheless, the modulation of NF-κB activation by PAR2 could have important implications for lung inflammation. For example, the suppression of NF-κB-dependent transcription by IL-4-induced activation of STAT6 reduced pro-inflammatory responses, including diminished activation of the inflammasome and decreased production of IL-1β [62, 63]. In addition, NF-κB activation promoted myofibroblast differentiation and exacerbated bleomycin-induced pulmonary fibrosis in mice [64]. Notably, exposure of lung fibroblasts to MWCNTs induced the phosphorylation and translocation of NF-κB and resulted in up-regulation of genes encoding pro-fibrotic mediators tissue inhibitor of metalloproteinase 1 (TIMP1) and osteopontin (OPN) [65].

The MWCNTs that we used in our present study (NC7000, Nanocyl, Inc.) are not necessarily representative of all MWCNTs that have been investigated regarding allergic lung disease. These MWCNTs were chosen since they represent a type of commercially available nanomaterial (Nanocyl.com) and can be further purified and functionalized to alter their toxicologic properties [25]. In our investigation, NC7000 MWCNTs alone induced a neutrophilic lung inflammatory response but not eosinophilic inflammation, and yet exacerbated the eosinophilic lung response induced by low doses of HDM extract. In contrast, others have shown that certain types of MWCNTs can induce an allergic lung inflammatory response alone characterized by eosinophilic lung inflammation, mucous cell metaplasia, and STAT-6 activation [13, 66–68]. For example, Carvalho et al., showed that eosinophilic lung inflammation in mice correlated with high nickel content in FA21 MWCNTs [13]. Notably, the NC7000 MWCNTs used in the present study do not contain nickel, but rather contain residual aluminum, cobalt and iron that were used as catalysts during synthesis (Additional file 1: Table S1). The rigid fiber-like shape of MWCNTs has also been correlated with direct asthma-like effects. For example, Rydman et al., reported that rod-like MWCNTs (Mitsui-7) directly cause eosinophilia in the lungs of mice [67]. We also previously reported that the rigidity of the MWCNTs can profoundly impact the host immune system [68]. Specifically, rigid, rod-like MWCNTs stimulated an allergic immune response in the lungs of mice, whereas tangled MWCNTs similar to the NC7000 used in the present study, did not. Finally, NC7000 MWCNTs are relatively thin compared to Mitsui-7 MWCNTs (12 nm vs ~ 50 nm, respectively), allowing for complete phagocytosis by macrophages, whereas Mitsui-7 MWCNTs cause frustrated phagocytosis in macrophages [68]. Therefore, it is important to consider the physicochemical properties of MWCNTs that trigger the host immune response.

While our findings show that MWCNTs exacerbate allergic lung disease in mice, it has yet to be determined whether MWCNTs exacerbate asthma in humans. The highest exposures to MWCNTs likely occur in occupational settings where these nanomaterials are manufactured and handled. For example, Lee et al. [69] were able to detect MWCNTs in the air of the manufacturing plants ranging from 0.002 to 24.9 µg/m^3^. Dahm et al. [70] reported that MWCNTs were found at concentrations up to 1050 µg/m^3^ during secondary manufacturing processes such as packaging and bagging of the nanotubes. Further epidemiological data is needed to assess whether workers in these occupational settings have a higher incidence of asthma or more severe asthma.

## Conclusion

The current study aimed to elucidate mechanisms through which nanoparticles exacerbate asthma by investigating the role of PAR2 in MWCNT-induced amplification of allergic lung disease in mice. Our findings demonstrated that MWCNTs produce strong pro-inflammatory and pro-fibrotic adjuvant effects in the presence of relatively low HDM extract doses that alone do not cause lung inflammation. Importantly, we found that PAR2 plays a role in airway fibrogenesis but not eosinophilic lung inflammation. The increasing demand for MWCNTs could increase the likelihood of occupational and consumer exposures, which in turn might lead to an increased risk for the exacerbation of allergic asthma in exposed individuals.

### Supplementary Information

**Additional file 1**: **Table S1**. Physicochemical characteristics of multi-walled carbon nanotubes.

**Additional file 2**: **Fig. S1**. Hematoxylin and eosin-stained lung tissue section showing eosinophilic granulomatous lesions containing MWCNTs in wild type mouse lung following exposure to HDM extract and MWCNTs by oropharyngeal aspiration.

**Additional file 3**: **Fig. S2**. Example of genotyping results of wild type and *Par2* KO mice used in this study.

**Additional file 4**: **Fig. S3**. CXCL-1 and CCL2 protein levels in BALF measured by ELISA.

**Additional file 5**: **Fig. S4**. Representative Alcian blue PAS-stained lung sections from WT and *Par2* KO mice showing mucous cell metaplasia after exposure to HDM extract and MWCNTs along with quantitative morphometry of all WT and *Par2* KO mice used in this study.

**Additional file 6**: **Fig. S5**. Quantitative morphometry of eosinophilic granulomas containing MWCNT in wild type and Par-2 KO mice.

**Additional file 7**: **Fig. S6**. Western blotting results on the lung tissue from 5 mice per group for each genotype that were captured using Amersham Imager 680. Cropped versions of these data were compiled and are shown in Fig. 4.

**Additional file 8**: **Fig. S7**. Semi-quantitative analysis of ARG-1 immunohistochemistry derived from QuPath software.

## Data Availability

Datasets generated for this study are available from the corresponding author upon reasonable request.

## Abbreviations

HDM: House dust mite
PM: Particulate matter
AHR: Airway hyper-responsiveness
BALF: Bronchoalveolar lavage fluid
LDH: Lactate dehydrogenase
PAR2: Protease-activated receptor-2
MWCNT: Multi-walled carbon nanotube
ENM: Engineered nanomaterial
CXCL1: C-X-C motif chemokine ligand 1
CCL2: C-C motif chemokine ligand 2
CCL11: Eotaxin
IL-6: Interleukin-6
OPN: Osteopontin
TGF-β1: Transforming growth factor-β1
TIMP-1: Tissue inhibitor of metalloproteinase 1
ARG-1: Arginase-1
NFκB: Nuclear factor κB
STAT-6: Signal transducer and activator of transcription 6
SMAD2/3: Suppressor of mothers against decapentaplegic 2/3
