# Supplementary material for: Role of the protease-activated receptor-2 (PAR2) in the exacerbation of house dust mite-induced murine allergic lung disease by multi-walled carbon nanotubes
Source: Part Fibre Toxicol. 2023 Aug 14;20:32. doi: 10.1186/s12989-023-00538-6 (PMC10424461; doi:10.1186/s12989-023-00538-6)
Supplement: Supplementary file 1 — Additional file 1: Table S1. Physicochemical characteristics of multi-walled carbon nanotubes. [file 12989_2023_538_MOESM1_ESM.pdf]

Additional File 1

Table S1. Selected physicochemical characterization of NC7000 MWCNTs[1].

**Amorphous Carbon**	4.68%
**Carbon**[2]	92.05% (EDX); 97.8% (XPS)
**Oxygen**	3.19% (EDX); 1.4 (XPS)
**Trace metals (ICP-MS)**<br>**Al**<br>**TE**[3]	 4.43%<br>0.54%
**Avg. Diameter (TEM)**	12 nm
**Length (TEM, SEM)**	1,350 nm
**BET Surface Area**	24 m^2/g
**Pore volume**	61 µl/g

[1]Data from: Taylor-Just et al., 2020.
[2]Carbon measured by EDX (energy dispersive X-ray analysis) or XPS (X-ray Photoelectron spectroscopy).
[3]TE = transition elements (Co and Fe)
