# Supplementary material for: Role of the protease-activated receptor-2 (PAR2) in the exacerbation of house dust mite-induced murine allergic lung disease by multi-walled carbon nanotubes
Source: Part Fibre Toxicol. 2023 Aug 14;20:32. doi: 10.1186/s12989-023-00538-6 (PMC10424461; doi:10.1186/s12989-023-00538-6)
Supplement: Supplementary file 2 — Additional file 2: Fig. S1. Hematoxylin and eosin-stained lung tissue section showing eosinophilic granulomatous lesions containing MWCNTs in wild type mouse lung following exposure to HDM extract and MWCNTs by oropharyngeal aspiration. [file 12989_2023_538_MOESM2_ESM.pdf]

Additional File 2

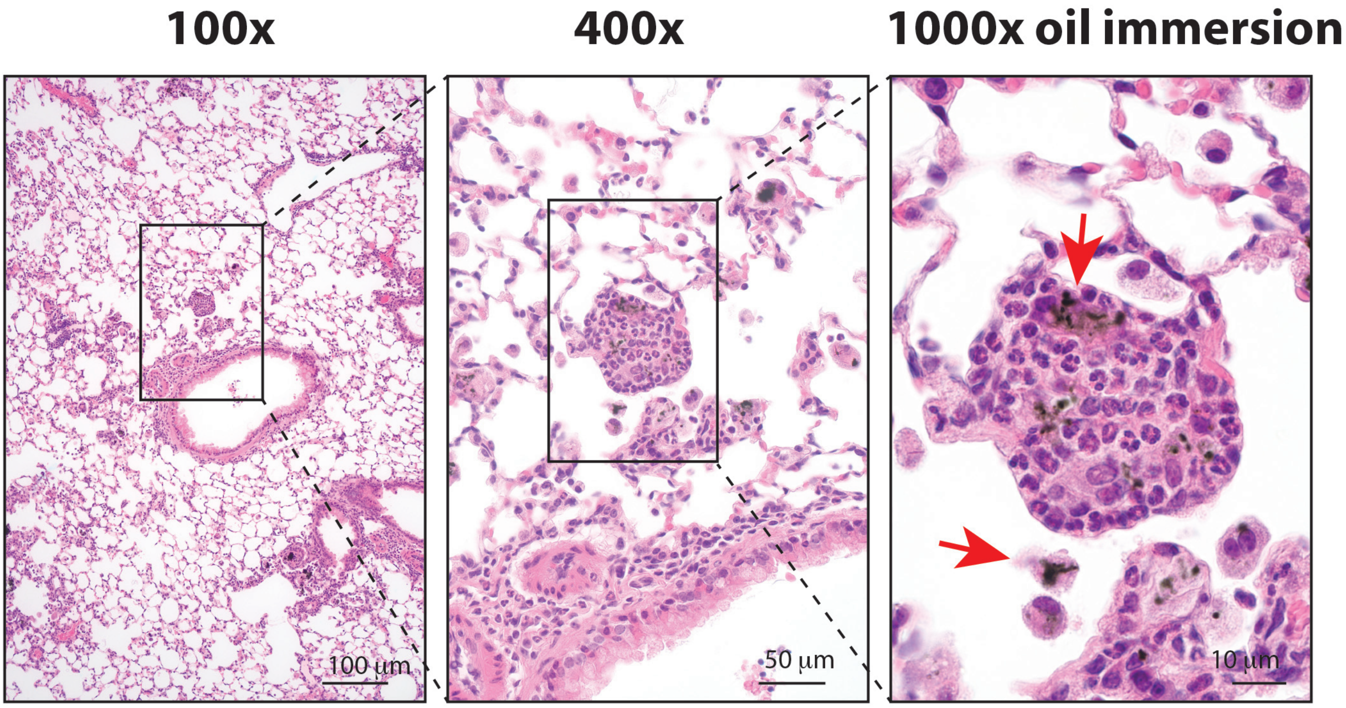

Fig. S1. Hematoxylin and eosin-stained lung tissue section showing eosinophilic granulomatous lesions containing MWCNTs in wild type mouse lung following exposure to HDM extract and MWCNTs by oropharyngeal aspiration.
Red arrows indicate MWCNTs.
