# Supplementary material for: Role of the protease-activated receptor-2 (PAR2) in the exacerbation of house dust mite-induced murine allergic lung disease by multi-walled carbon nanotubes
Source: Part Fibre Toxicol. 2023 Aug 14;20:32. doi: 10.1186/s12989-023-00538-6 (PMC10424461; doi:10.1186/s12989-023-00538-6)
Supplement: Supplementary file 3 — Additional file 3: Fig. S2. Example of genotyping results of wild type and Par2 KO mice used in this study. [file 12989_2023_538_MOESM3_ESM.pdf]

Additional File 3

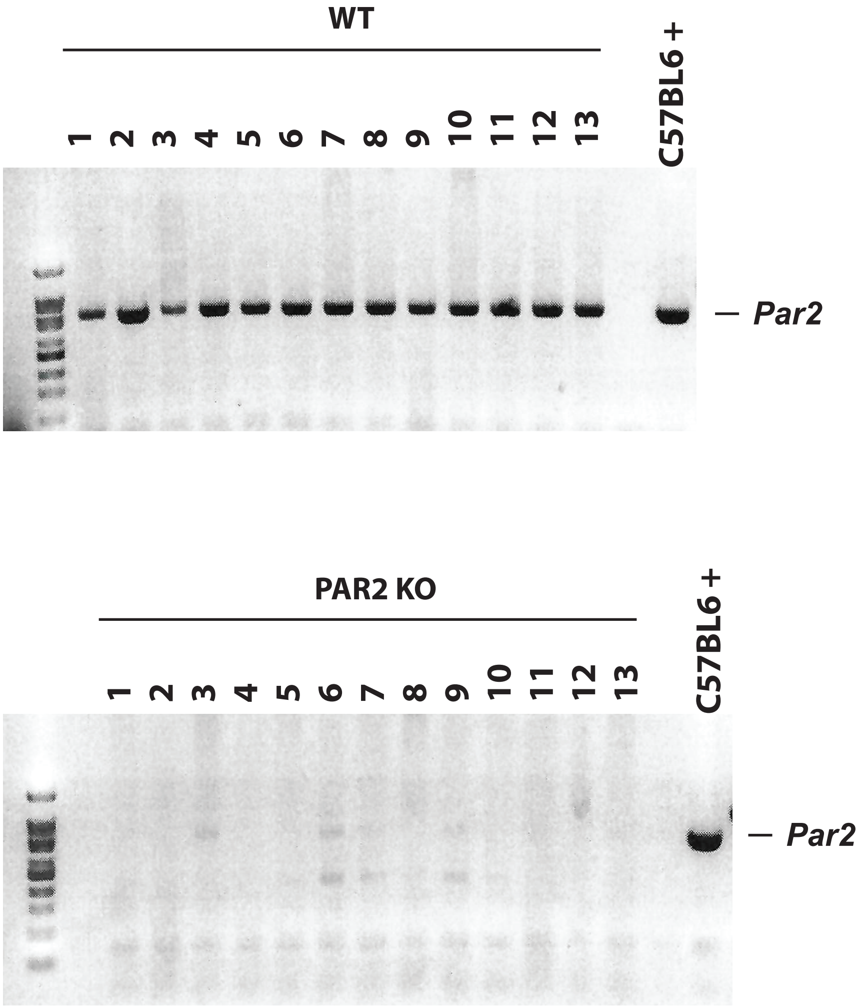

Fig. S2. Example of genotyping results of wild type (WT) and par2 KO mice used in this study. DNA from C57BL6 mice were used as a positive control.
