# Supplementary material for: Role of the protease-activated receptor-2 (PAR2) in the exacerbation of house dust mite-induced murine allergic lung disease by multi-walled carbon nanotubes
Source: Part Fibre Toxicol. 2023 Aug 14;20:32. doi: 10.1186/s12989-023-00538-6 (PMC10424461; doi:10.1186/s12989-023-00538-6)
Supplement: Supplementary file 4 — Additional file 4: Fig. S3. CXCL-1 and CCL2 protein levels in BALF measured by ELISA. [file 12989_2023_538_MOESM4_ESM.pdf]

Additional File 4

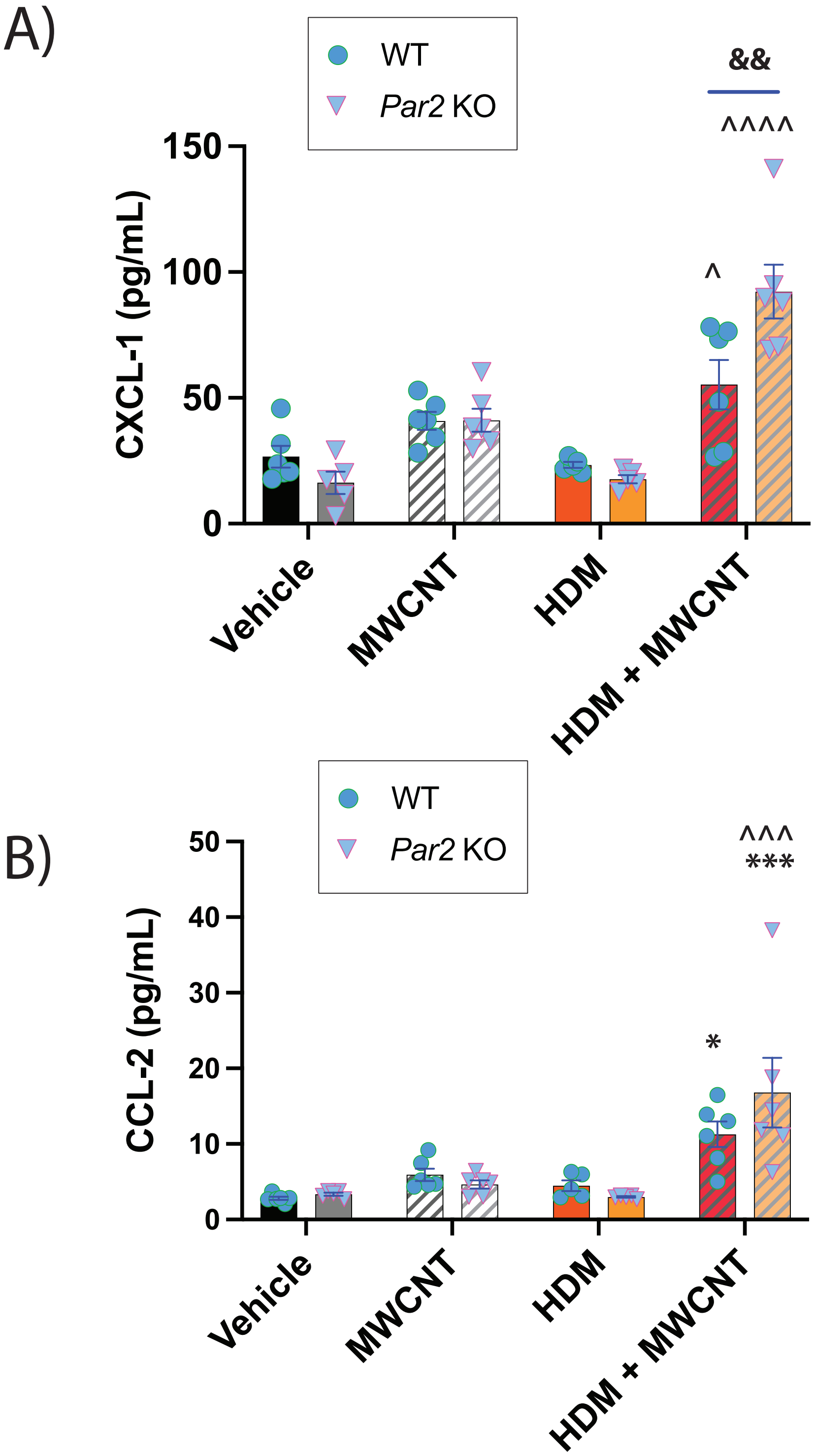

Fig. S3. Protein levels of CXCL-1 (**A**) and CCL-2 (**B**) in BALF from WT and par2 KO mice. *p,0.05 compared to vehicle, ***p,0.001 compared to vehicle, ^p<0.05 compared to HDM, ^^^p,0.001 compared to HDM, &&p,0.01 between genotypes.
