# Supplementary material for: Role of the protease-activated receptor-2 (PAR2) in the exacerbation of house dust mite-induced murine allergic lung disease by multi-walled carbon nanotubes
Source: Part Fibre Toxicol. 2023 Aug 14;20:32. doi: 10.1186/s12989-023-00538-6 (PMC10424461; doi:10.1186/s12989-023-00538-6)
Supplement: Supplementary file 5 — Additional file 5: Fig. S4. Representative Alcian blue PAS-stained lung sections from WT and Par2 KO mice showing mucous cell metaplasia after exposure to HDM extract and MWCNTs along with quantitative morphometry of all WT and Par2 KO mice used in this study. [file 12989_2023_538_MOESM5_ESM.pdf]

Additional File 5

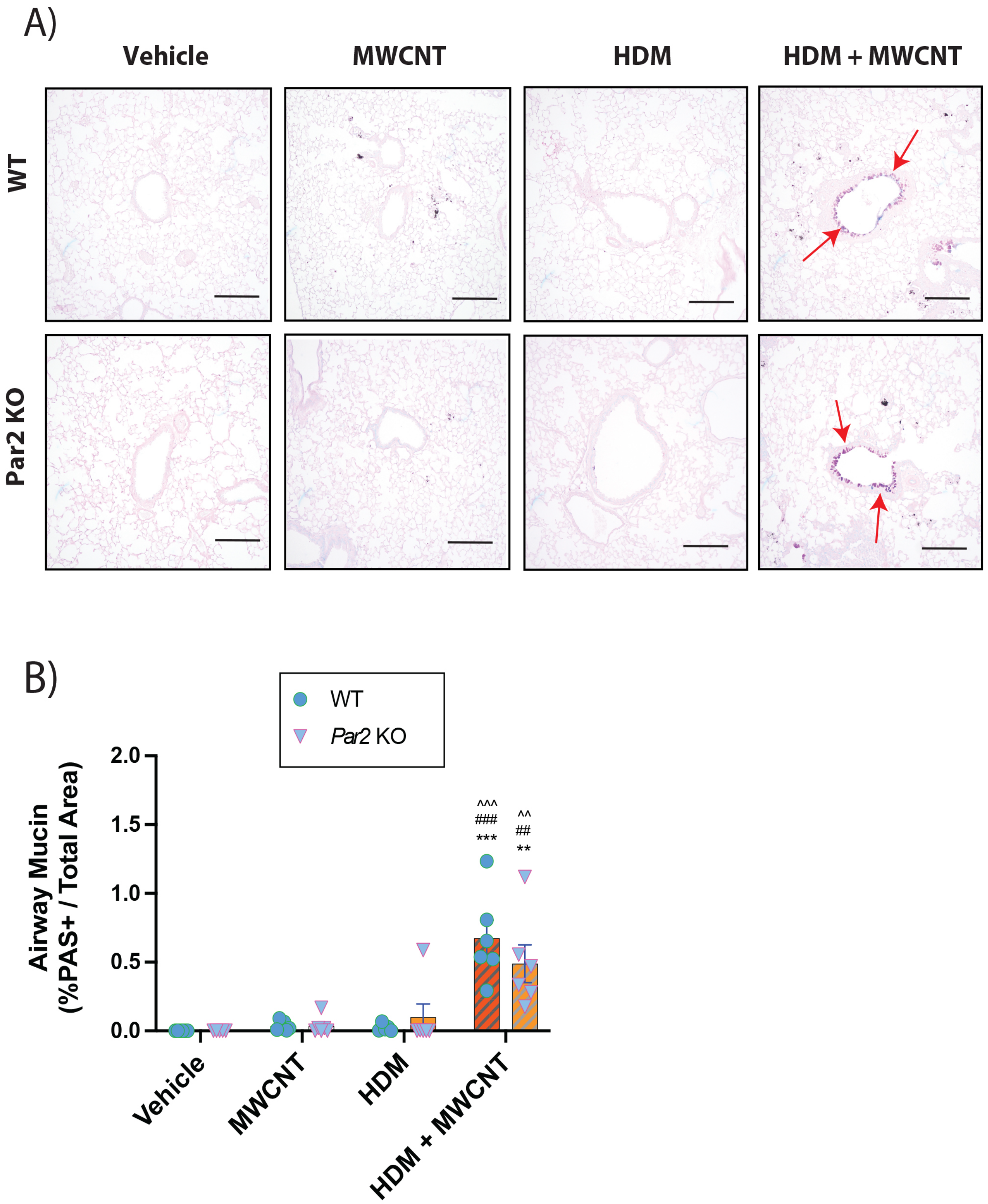

Fig. S4. Panel A: Alcian blue PAS staining of lung tissue from WT and Par2 KO mice showing mucous cell metaplasia after exposure to HDM extract and MWCNTs in both genotypes. Red arrows indicate mucous cell metaplasia. Magnification bars = 100 μm. Panel B: Quantitative morphometry of airway mucin in WT and Par2 KO mice.
