# Supplementary material for: Role of the protease-activated receptor-2 (PAR2) in the exacerbation of house dust mite-induced murine allergic lung disease by multi-walled carbon nanotubes
Source: Part Fibre Toxicol. 2023 Aug 14;20:32. doi: 10.1186/s12989-023-00538-6 (PMC10424461; doi:10.1186/s12989-023-00538-6)
Supplement: Supplementary file 6 — Additional file 6: Fig. S5. Quantitative morphometry of eosinophilic granulomas containing MWCNT in wild type and Par-2 KO mice. [file 12989_2023_538_MOESM6_ESM.pdf]

Additional File 6

A)

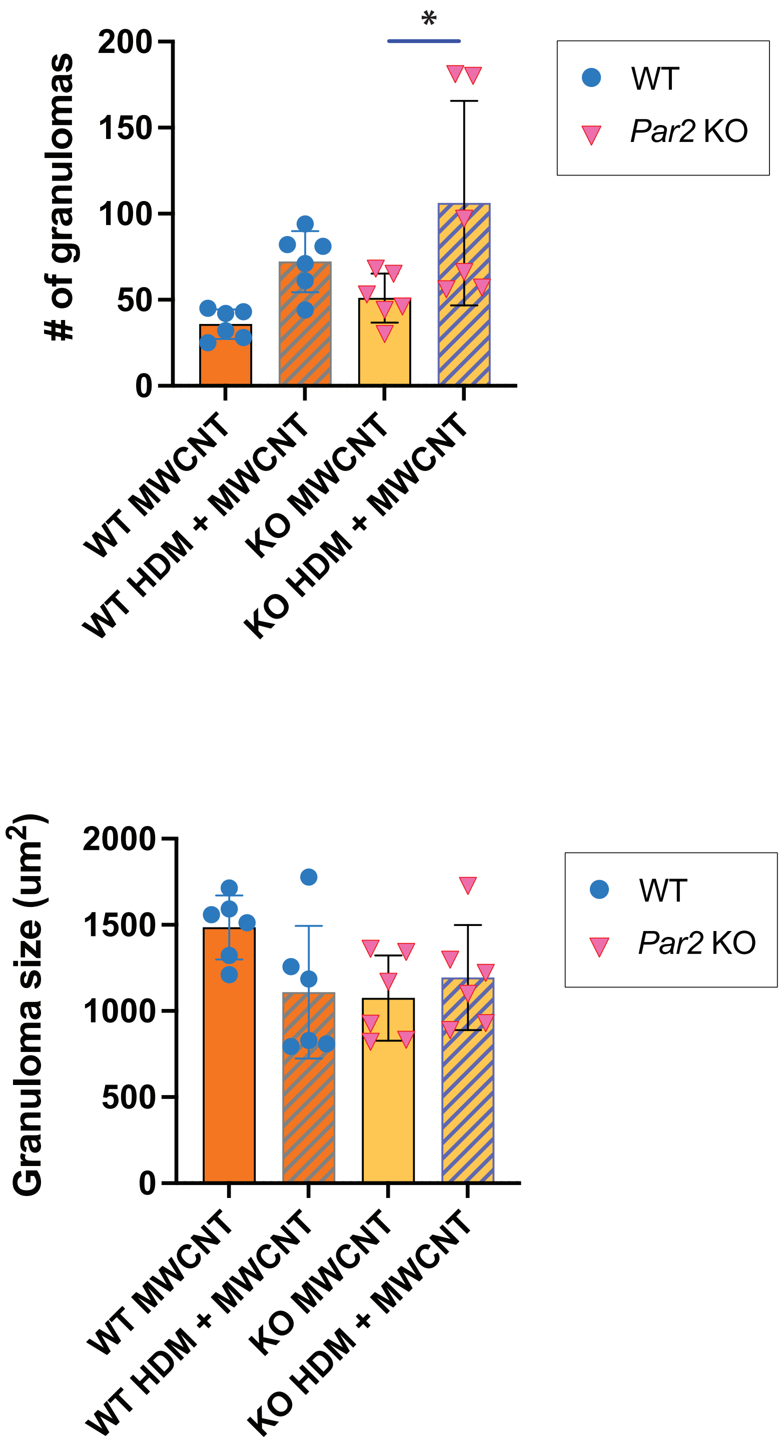

Fig. S5. Quantitiative morphometry of eosinophilic granulomas containing MWCNT in wild type and Par2 KO mice. (**A**) Numbers of granulomas per lung section. *p<0.05 compared to MWCNT alone. (**B**) Average size of granulomas.
