# Supplementary material for: Role of the protease-activated receptor-2 (PAR2) in the exacerbation of house dust mite-induced murine allergic lung disease by multi-walled carbon nanotubes
Source: Part Fibre Toxicol. 2023 Aug 14;20:32. doi: 10.1186/s12989-023-00538-6 (PMC10424461; doi:10.1186/s12989-023-00538-6)
Supplement: Supplementary file 7 — Additional file 7: Fig. S6. Western blotting results on the lung tissue from 5 mice per group for each genotype that were captured using Amersham Imager 680. Cropped versions of these data were compiled and are shown in Fig. 4. [file 12989_2023_538_MOESM7_ESM.pdf]

Additional File 7

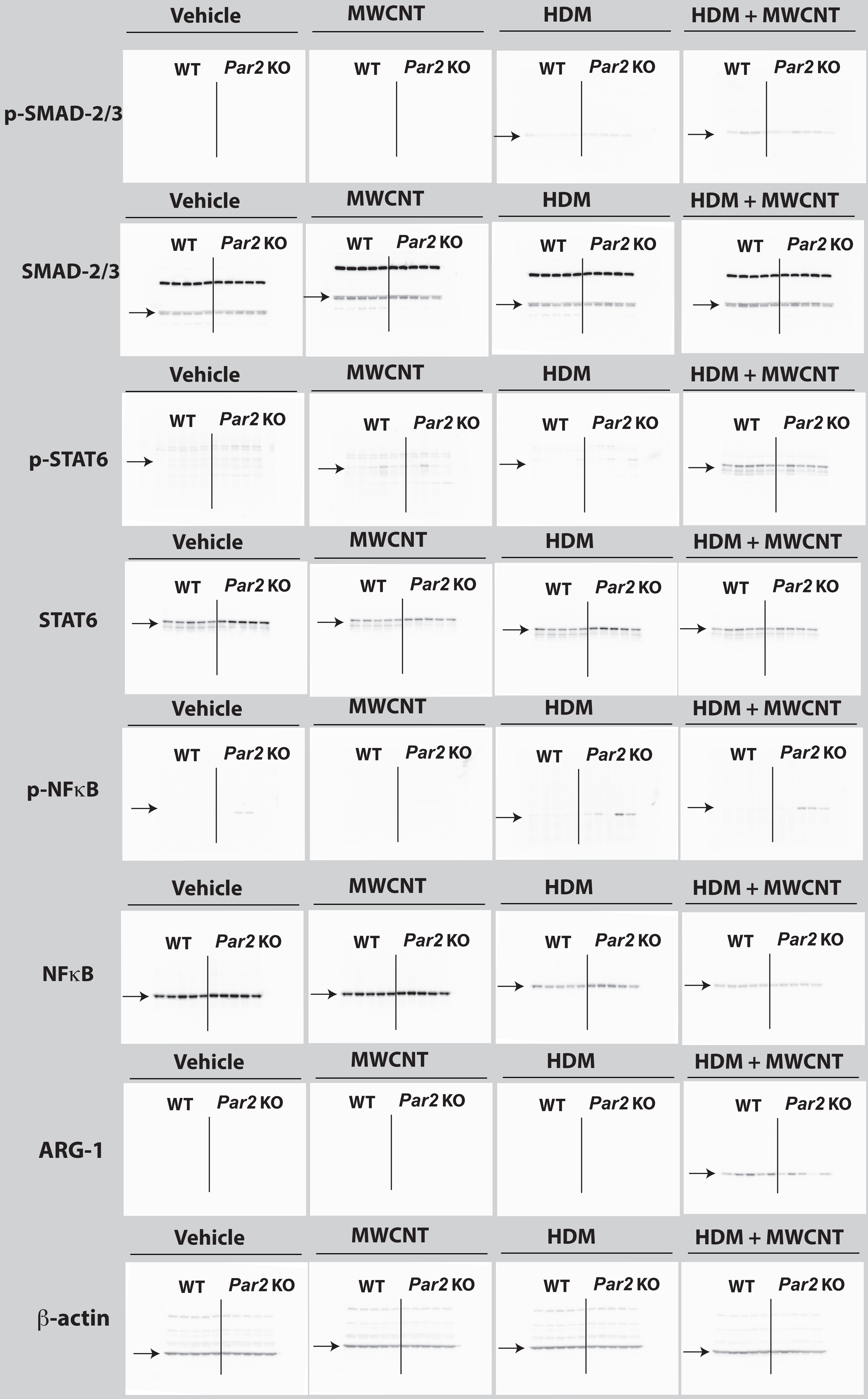

Fig. S6. Western blotting results on the lung tissue from wild type (WT) or Par2 KO mice (5 mice per group) for each of the four treatment groups captured using Amersham Imager 680 (see Methods). Arrows indicate the position of each specific protein. Cropped versioins of western blots are shown in Fig. 4.
