# Supplementary material for: Role of the protease-activated receptor-2 (PAR2) in the exacerbation of house dust mite-induced murine allergic lung disease by multi-walled carbon nanotubes
Source: Part Fibre Toxicol. 2023 Aug 14;20:32. doi: 10.1186/s12989-023-00538-6 (PMC10424461; doi:10.1186/s12989-023-00538-6)
Supplement: Supplementary file 8 — Additional file 8: Fig. S7. Semi-quantitative analysis of ARG-1 immunohistochemistry derived from QuPath software. [file 12989_2023_538_MOESM8_ESM.pdf]

Additional File 8

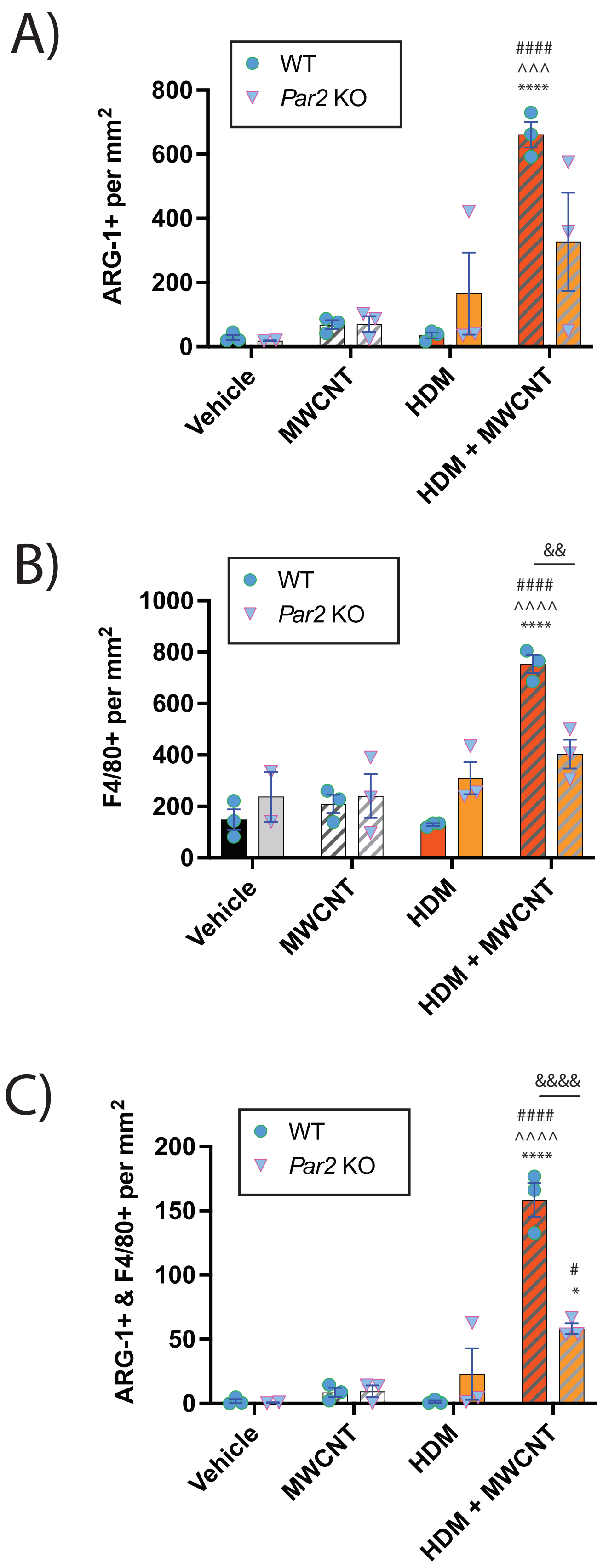

Fig. S7 Semi-quantitative analysis of ARG-1 immunohistochemistry derived from QuPath software. **(A)** the number of ARG-1+ cells per mm^2 lung tissue sections per animal. **(B)** Percent of cells that were DAPI+ and ARG-1+, and **(C)** Percent of cells that were ARG-1+ and F4/80+. The data represent 3 animals per group for each genotype.
